# Microscopic extrathyroid extension in papillary thyroid carcinoma: impact on response to therapy

**DOI:** 10.20945/2359-3997000000210

**Published:** 2020-03-18

**Authors:** Bernardo Marques, Raquel G. Martins, Joana Couto, Jacinta Santos, Teresa Martins, Fernando Rodrigues

**Affiliations:** 1 Endocrinology Department Instituto Português de Oncologia de Coimbra Francisco Gentil EPE Coimbra Portugal Endocrinology Department, Instituto Português de Oncologia de Coimbra Francisco Gentil, EPE, Coimbra, Portugal

**Keywords:** Thyroid cancer, management, papillary thyroid cancer, radioiodine therapy, relapse predictors

## Abstract

**Objectives:**

Evaluate the impact of microscopic extrathyroid extension (MEE) on outcome and therapy response in patients with cT1 and cT2 papillary thyroid carcinoma (PTC).

**Subjects and methods:**

Retrospective study of 970 consecutive patients, who underwent surgery for PTC between 2000 and 2016. All patients had: tumours ≤ 4 cm, apparent complete tumour resection, without clinically apparent lymph node or distant metastasis at diagnosis and nonaggressive histologic variant.

**Results:**

Based on the finding of MEE, 175 (18.0%) patients were upstaged to T3. They were older (53.9 *versus* 50.6 years; *P* = 0.004) and were more prone to have multifocal tumours (38.2% *versus* 24.8%; *P* = 0.001). Radioiodine ablation therapy (RAI) was administered more often to MEE patients (92% *versus* 40.5%; *P* < 0.001), as well as prophylactic lymph node resection (35.4% *versus* 28.6%, *P* = 0.048). They were more likely to have biochemical incomplete response (4% *versus* 0.3%; *P* = 0.03) at the end of the follow-up period. There was no significant association between MEE and recurrence rate, persistence of disease or disease-specific mortality.

**Conclusion:**

These results support the changes made to the latest edition of the TNM staging system, regarding MEE. Although incomplete biochemical response is more common in these patients, it does not seem to affect their prognosis.

## INTRODUCTION

Well-differentiated thyroid cancer (WDTC) is, generally, a disease with good prognosis. Its management continues to move towards a more personalized approach and initial risk stratification is an important tool used to guide early initial therapy and follow-up, such as extent of thyroid surgery, RAI and levothyroxine therapy (1-4). These initial risk estimates are further modified based on each patient’s response to therapy and the biological behaviour of the disease – an approach called dynamic risk stratification. This approach can lead to changes in the long term management, namely degree of TSH suppression, frequency of follow-up and additional imaging exams (1-3).

An important factor predictive of outcome has been considered to be extrathyroid extension (ETE) (1,5-8). However, recent studies suggest that gross ETE is of more significance than MEE (5-7,9-11). Until recently, according to the TNM staging system, the finding of MEE by a pathologist, in an otherwise clinical T1 and T2 tumour (≤ 4 cm), resulted in an upstage to pT3 and, in patients older than 45 years old, this finding leads to an upstage to stage III, suggesting that these patients had a poorer survival. According to the 2015 American Thyroid Association (ATA) guidelines on WDTC risk stratification system, these patients are regarded as “intermediate risk of disease recurrence”, and the majority is treated with total thyroidectomy and RAI (1). On the other hand, there is no data supporting the finding of MEE as a risk factor for disease recurrence or mortality and recent studies suggest that MEE may have little importance in tumours smaller than 4cm (T1 and T2) (9,11). Furthermore, changes have been made on the definition of T3 in the 8^th^ edition of the TNM staging system (2016). In this latest edition, tumours larger than 4 cm limited to the thyroid are classified as T3a, and tumours of any size with gross ETE (involving only strap muscles) are classified as T3b. Unlike previous editions of the TNM staging system, MEE is not used as a risk factor for staging and does not imply an upstage to pT3 or stage III (12).

However, there are no so studies on the impact of MEE in response to therapy. This study aims to evaluate the impact of MEE on outcome in patients with otherwise T1 and T2 PTC and its effect on therapy response.

## SUBJECTS AND METHODS

Nine hundred and seventy consecutive patients (970) were identified from our institutional database, who underwent surgery for clinically T1 and T2 papillary thyroid carcinoma between 2000 and 2016. The median follow-up period was 7.5 (4.8-10.7) years. Patient demographics, extent of thyroid surgery, details of pathology (histology, tumour size and presence of MEE), surgery complications, levothyroxine supression therapy and use of RAI therapy were recorded from patients’ charts.

MEE was described as tumour that had breached the thyroid capsule and microscopic examination showed it to be invading the first soft tissue immediately beyond the capsule. On the other hand, gross ETE was defined as ETE which was noticed intraoperatively or on naked-eye examination and later confirmed by microscopy. Recurrence was defined as detection of disease after a period when it wasn’t detectable and persistence was defined as detectable disease at the end of the follow-up period. Both were assessed based on clinical examination, thyroglobulin level and confirmed afterwards by imaging, cytological and histopathological examination. Response to therapy in patients who underwent total thyroidectomy and RAI was defined according to the 2015 ATA guidelines on WDTC (1). However, this system is not yet valited for WDTC patients who underwent lobectomy or patients not treated with RAI. For these cases we assessed response to therapy, based on the study performed by DP Momesso and cols. (13) (Table 1).

**Table 1:** Response to therapy assessment definitions

	TT with RAI	TT without RAI	Lobectomy
**Excellent response:** no clinical, biochemical or structural evidence of disease.	Supressed Tg < 0.2 ng/mL or TSH-stimulated Tg < 1 ng/mL	Supressed Tg < 0.2 ng/mL or TSH-stimulated Tg < 2 ng/mL	Supressed Tg < 30 ng/mL
**Biochemical incomplete response:** abnormally elevated Tg or or rising anti-Tg antibody levels in the absence of localizable disease.	Supressed Tg ≥ 1 ng/mL or TSH-stimulated Tg ≥ 10 ng/mL	Supressed Tg ≥ 5 ng/mL or TSH-stimulated Tg ≥ 10 ng/mL	Supressed Tg > 30 ng/mL
**Structural incomplete response:** persistent or newly identified loco-regional or distant metastases	Regardless of Tg or anti-Tg antibody levels	Regardless of Tg or anti-Tg antibody levels	Regardless of Tg or anti-Tg antibody levels
**Indeterminate response:** nonspecific findings that cannot be confidently classified as either benign or malignant.	Nonstimulated Tg 0.2-1 ng/mL or TSH-stimulated Tg 1-10 ng/mL	Nonstimulated Tg 0.2-5 ng/mL or TSH-stimulated Tg 2-10 ng/mL	

TT: total thyroidectomy; Tg: thyroglobulin.

Patients who were classified as T3 based on the size of the tumour (larger than 4 cm) were excluded, along with patients with evidence of gross ETE. All patients had tumours smaller than 4 cm, apparent complete tumour resection and nonaggressive histologic variant (we included the conventional and follicular variants). None of the patients had clinically detectable lymph node or distant metastasis at diagnosis, as well as positive nodes after central compartment neck dissection.

Statistical analysis was performed using SPSS Statistics 23.0. The association between variables was assessed using chi-square, Student’s t-tests and Fisher’s exact test.

## RESULTS

From this cohort, 175 (18.0%) patients were classified as T3 based on the finding of MEE and 795 (82.0%) were classified as T1 (N = 621; 64.0%) or T2 (N = 174; 17.39%). Based on the 8^th^ edition of the TNM staging system for WDTC, 141 MEE patients would be downgraded to T1 (14.5%) and 34 to T2 (3.5%). Regarding the staging changes, 87 MEE patients (49.7%) would be downstaged to stage II and 50 patients (28.6%) to stage I.

The male to female ratio was 1:6 and there were no differences regarding gender and presence of MEE (*P* = 0.310). Patients with MEE were older than patients without MEE (mean age = 53.9 *versus* 50.6 years, *P* = 0.004) and were more prone to have multifocal tumours (38.2% *versus* 24.8%; *P* = 0.001). Mean tumour size was similar between both groups (14.8 mm *versus* 15.1 mm). Regarding histological subtypes, there were 810 cases (83.5%) of classic PTC and 160 follicular variant PTC (16.5%), with classic PTC being the most common histological subtype in both groups (88.0% *versus* 82.6%). We proceeded to review the cases of encapsulated follicular variant PTC in order to determine if they were Noninvasive Follicular Thyroid Neoplasm with Papillary-like Nuclear Features (NIFTP) cases. The cases in which NIFTP was confirmed were excluded from the sample. These results are shown in Table 2.

**Table 2:** Patients and tumours’ characteristics, stratified by MEE

Variable		Microscopic extra-thyroid extension		*P*

		Present N = 175	Absent N = 795	
Age – years (mean ± SD)		53.9 ± 11.7	50.6 ± 14.7	**0.004**
Gender – N (%)				
Male		23 (13.1%)	129 (16.2%)	NSS
Female		152 (86.9%)	666 (83.8%)	
T1 (tumour size ≤ 20 mm)		141 (80.6%)	621 (78.1%)	NSS
T2 (tumour size 21-40 mm)		34 (19.4%)	174 (21.9%)	NSS
Mean tumour size (mm) ± SD		14.8 ± 8.1	15.1 ± 10.3	NSS
Histological subtype				
Classic papillary		151 (86.3%)	656 (82.6%)	NSS
Follicular variant		24 (13.7%)	139 (17.4%)	NSS
Multifocalilty – N (%)		67 (38.2%)	197 (24.8%)	0.001
Median follow-up time – years (IQR)		7.0 (4.5-9.9)	7.6 (4.8-10.1)	NSS

SD: Standard deviaton; N: number; RAI: radioiodine ablation therapy; NSS: non statistical significant; IQR: interquartile range.

There was no significant association between MEE and extent of thyroid surgery, as total thyroidectomy was performed in almost every patient in both groups (94.3% of T3 patients *versus* 90.9% of T1/T2 patients). However, prophylactic central neck compartment dissection was performed more often to T3 patients (35.4% *versus* 28.6%, *P* = 0.048). Therapy with RAI was given more frequently in MEE patients than patients without MEE (92% *versus* 40.5%; *P* < 0.001) and with a higher mean cumulative dose (105.6 mCi *versus* 79.2 mCi; *P* = 0.002). Patients with MEE were also more likely to perform an additional radioiodine treatment (4.6% *versus* 1.3%; *P* = 0.036).

At the end of the follow-up period, the proportion of patients under levothyroxine suppressive therapy was similar in both groups (22.3% *versus* 26.1%). Additional surgery was performed in patients with persistent or recurrence of disease at a similar rate between MEE patients and T1-T2 patients (2.3% *versus* 2.0%). These results are shown in Table 3.

**Table 3:** Patients’ treatment, stratified by MEE

Treatment modality	Microscopic Extra-Thyroid Extension		*P*

	Present N = 175	Absent N = 795	
RAI – N (%)	161 (92.0%)	322 (40.5%)	< 0.001
RAI activity level 30-49 mCi	0 (0%)	3 (0.9%)	NSS
RAI activity level 50-99 mCi	62 (38.5%)	205 (63.6%)	< 0.001
RAI activity level 100-149 mCi	90 (55.9%)	102 (31.7%)	< 0.001
RAI activity level ≥ 150 mCi	9 (5.6%)	12 (3.7%)	NSS
Mean cumulative dose of RAI (mCi) ± SD	105.6 ± 54.3	79.2 ± 32.3	0.002
Total thyroidectomy – N (%)	165 (94.3%)	723 (90.9%)	NSS
Prophylactic lymph node resection – N (%)	62 (35.4%)	227 (28.6%)	0.048
Levothyroxine supressive therapy	39 (22.3%)	208 (26.1%)	NSS
TSH 0.1-0.45 UI/mL – N (%)	28 (71.8%)	151 (72.6%)	NSS
TSH <0.1 UI/mL – N (%)	11 (28.2%)	57 (27.4 %)	NSS
Additional surgery	4 (2.3%)	16 (2.0%)	NSS
Additional radioiodine treatment	9 (5.6%)	10 (1.3%)	0.036
Radioiodine activity level 100-149 mCi	2 (22.2%)	2 (20%)	NSS
Radioiodine activity level ≥ 150 mCi	7 (77.8%)	8 (80%)	NSS

SD: Standard deviaton; N: number; RAI: radioiodine ablation therapy; NSS: non statistical significant.

Regarding response to therapy, 90.1% of patients showed excellent response at the end of the follow-up period, with similar rates comparing patients with MEE and T1/T2 patients (89.8% *versus* 92.9%). Excellent response to therapy rate was also similar between MEE patients who underwent prophylactic central neck compartment dissection and those who did not (93.2% *versus* 92.8%). Moreover, there were no differences between both groups regarding undetermined response to therapy (6.5% *versus* 4.7%) and structural incomplete response to therapy rates (0.7% *versus* 2.7%). However, MEE patients who underwent RAI therapy had a higher rate of excellent response to therapy than those who did not (91.9% *versus* 71.4%; *P* = 0.022). Moreover, MEE patients were more prone to show biochemical incomplete response to therapy (4% *versus* 0.4%; *P* = 0.030), compared to T1/T2 patients.

At the end of the follow-up period, recurrence of disease was found in 22 patients (2.3%) and persistence was found in 14 patients (1.4%). There was no significant difference between both groups (with or without MEE) regarding recurrence (1.7% *versus* 2.3%) or persistence of disease (3.4% *versus* 0.8%). Six patients, all of them without MEE, had distant metastasis identified at the end of the follow-up period, and two of them also had cervical lymph node metastasis. No disease-specific mortality occurred in either group.

The rate of treatment complications, namely chronic hypoparathyroidism and vocal cord paralysis was similar between both groups (10.9% *versus* 10.6%) and (1.1% in both groups), respectively. These results are shown in Table 4.

**Table 4:** Response to therapy, outcomes ant treatment complications, stratified by MEE

Variable	Microscopic Extra-Thyroid Extension		*P*

	Present N = 175	Absent N = 795	
Excellent response – N (%)	157 (89.8%)	738 (92.9%)	NSS
Indeterminate response – N (%)	8 (4.7%)	50 (6.5%)	NSS
Biochemical incomplete response – N (%)	7 (4.0 %)	3 (0.4%)	0.030
Structural incomplete response – N (%)	0 (0%)	6 (0.6%)	NSS
Cervical metastases	0 (0%)	2 (0.25%)	NSS
Distant metastases	0 (0%)	6 (0.75%)	NSS
Recurrence – N (%)	3 (1.7%)	19 (2.3%)	NSS
Persistence of disease – N (%)	6 (3.4%)	8 (0.8%)	NSS
Disease-specific mortality – N (%)	0	0	
Chronic hypoparathyroidism	19 (10.9%)	84 (10.6%)	NSS
Vocal cord paralysis	2 (1.1%)	9 (1.1%)	NSS

N: number; NSS: non statistical significant.

## DISCUSSION

The current study describes the outcomes of a very large cohort of patients with cT1/T2N0 PTC, with a long follow-up period. One hundred and seventy five (18.0%) patients were classified as T3 based on the finding of MEE by the pathologist, despite having otherwise, tumours smaller than 4 cm. In this group, patients tended to be older and have multifocal tumours and they were in fact submitted to more aggressive treatments, namely prophylactic lymph node resection, higher doses of RAI and additional radioiodine treatments. Nonetheless, the outcomes and surgery complications between both groups were similar. Furthermore, despite having a higher rate of biochemical incomplete response, MEE showed no effect on recurrence and persistence of disease at the end of the follow-up period and the rate of excellent response to therapy was similar between both groups.

Other studies published on MEE as a prognostic factor in WDTC also support the changes made on the TNM staging system. Shin JH and cols reported on 332 patients with PTC and showed that MEE did not impact recurrence-free survival. Nixon IJ and cols. reported on 984 patients who underwent surgery for cT1/T2 PTC and also showed that patients’ outcomes were excellent and not affected by the finding of MEE (5,11). However, to our knowledge, there are no studies published on the impact of MEE on response to therapy.

As with most clinical studies of thyroid cancer, this is a retrospective study, which makes it difficult to avoid possible biases. Patients with MEE have been treated more aggressively over the years, as recommended when they were diagnosed with WDTC and this may have contributed to favorable outcomes, similar to those of patients without MEE (1,4,7). Nevertheless, excellent response to therapy rate was similar between MEE patients who underwent prophylactic central neck compartment dissection and those who did not, which shows that this procedure might not be useful in patients with MEE. The decision to perform prophylactic central neck compartment dissection was done heterogeneously and varied according to the surgical team and the hospital in which the patient had surgery. Thus, different protocols were used over the years and it’s challenging to perform a fair assessment about its true therapeutic utility. RAI therapy, however, may have an impact on response to therapy, as MEE patients who underwent it had a higher rate of excellent response, compared to MEE patients who did not. There were no differences regarding the other grades of response to therapy. However, in relation to this aspect, it might be difficult to draw any more conclusions because, in our cohort, only 14 patients with MEE (8%) did not perform RAI therapy post-operatively. We changed our institutional protocol soon after the 8th edition of the TNM staging system was published but previously, however, all patients with MEE, regardless of tumour size, were offered treatment with RAI. Nonetheless, when comparing these patients with the remaining patients who were not submitted to RAI and to MEE patients submitted to RAI, the outcomes are still similar between these groups. These results are shown in Table 5.

**Table 5:** Outcomes stratified by RAI treatment and presence of MEE

Treatment modality	RAI			No RAI			T3		

	T3 N = 161	T1-2 N = 322	*P*	T3 N = 14	T1-2 N = 473	*P*	RAI N = 161	No RAI N = 14	*P*
Recurrence – N (%)	2 (1.2%)	1 (0.3%)	NSS	1 (7.1%)	18 (3.8%)	NSS	2 (1.2%)	1 (7.1%)	NSS
Persistence of disease – N (%)	6 (3.4%)	8 (0.8%)	NSS	0 (0%)	0 (0%)	NSS	6 (3.4%)	0 (0%)	NSS

N: number; NSS: non statistical significant.

Another bias is related to the fact that patients with MEE were older than those without MEE, which may be expected to worse their outcome, but they were also more likely to be treated with RAI.

This is the first study that evaluates the impact of MEE finding in patient’s response to therapy, as well as outcome. MEE patients had a higher rate of incomplete biochemical response and those who underwent RAI therapy had a higher rate of excellent response to therapy. Despite this, MEE patients had similar outcomes compared to patients with intrathyroid tumours.

In conclusion, outcomes in patients with well differentiated clinically and grossly intrathyroid cancers are excellent and may not be significantly affected by the discovery of MEE. These patients may be effectively treated with a conservative management strategy in terms of surgical treatment, RAI and levothyroxine therapy and avoid potential adverse effects of more aggressive treatments. However, long-term follow-up in MEE patients treated conservatively is necessary in order to confirm that biochemical incomplete response will not affect these patients’ prognosis.
